# Supplementary material for: Inositol Phosphoryl Transferase, Ipt1, Is a Critical Determinant of Azole Resistance and Virulence Phenotypes in Candida glabrata
Source: J Fungi (Basel). 2022 Jun 21;8(7):651. doi: 10.3390/jof8070651 (PMC9322651; doi:10.3390/jof8070651)
Supplement: Supplementary file 1 [file jof-08-00651-s001.zip › jof-1775877-supplementary/jof-1775877-supplementary.pdf]

Legends of supplementary data

Table S1. List of strains used in the study.

Figure S1. Drug susceptibility analysis of WT, *ΔCgipt1* and *ΔCgipt1::IPT1* strains using the spot assay. (A-B) Drug susceptibility to KTZ, MCZ, CTZ, FLC, ITR and PCZ and other xenobiotics was determined by spot assay.

Figure S2. Drug susceptibility analysis of WT and *ΔCgskn1* strains. Drug susceptibility was determined by spot assay.

Figure S3. PCA analysis of lipid species dataset of WT and *ΔCgipt1*. (A) Principal components 1 and 2 account for as much as 88% variance in the datasets. (B) PCA loading plot representing the contribution to individual lipid species to the overall variance is shown.

Supplementary sheet S1. Comparative lipidomic data of WT, Δ*Cgipt1* and Δ*Cgskn1* strains. Data represented in nmol per mg lipid dry wt (n=3).
