# Supplementary material for: Inositol Phosphoryl Transferase, Ipt1, Is a Critical Determinant of Azole Resistance and Virulence Phenotypes in Candida glabrata
Source: J Fungi (Basel). 2022 Jun 21;8(7):651. doi: 10.3390/jof8070651 (PMC9322651; doi:10.3390/jof8070651)
Supplement: Supplementary file 1 [file jof-08-00651-s001.zip › jof-1775877-supplementary/Table S1.pdf]

Table S1

List of strains used in the study

Strain	Genotype	Source
WT	BG2	Lab stock
ΔCgipt1	*ΔCgipt1::NAT1*	This study
ΔCgskn1	*ΔCgskn1::NAT1*	This study
Cgipt1/Cgskn1ΔΔ	BG14, ΔCgipt1::FRT/ΔCgskn1::NAT1	This study
ΔCgipt1::*IPT1*	ΔCgipt1::nat1, with plasmid pGRB2.3_hph_ipt1	This study
