# Supplementary figures and images for: Inositol Phosphoryl Transferase, Ipt1, Is a Critical Determinant of Azole Resistance and Virulence Phenotypes in Candida glabrata
Source: J Fungi (Basel). 2022 Jun 21;8(7):651. doi: 10.3390/jof8070651 (PMC9322651; doi:10.3390/jof8070651)

**Figure S1**

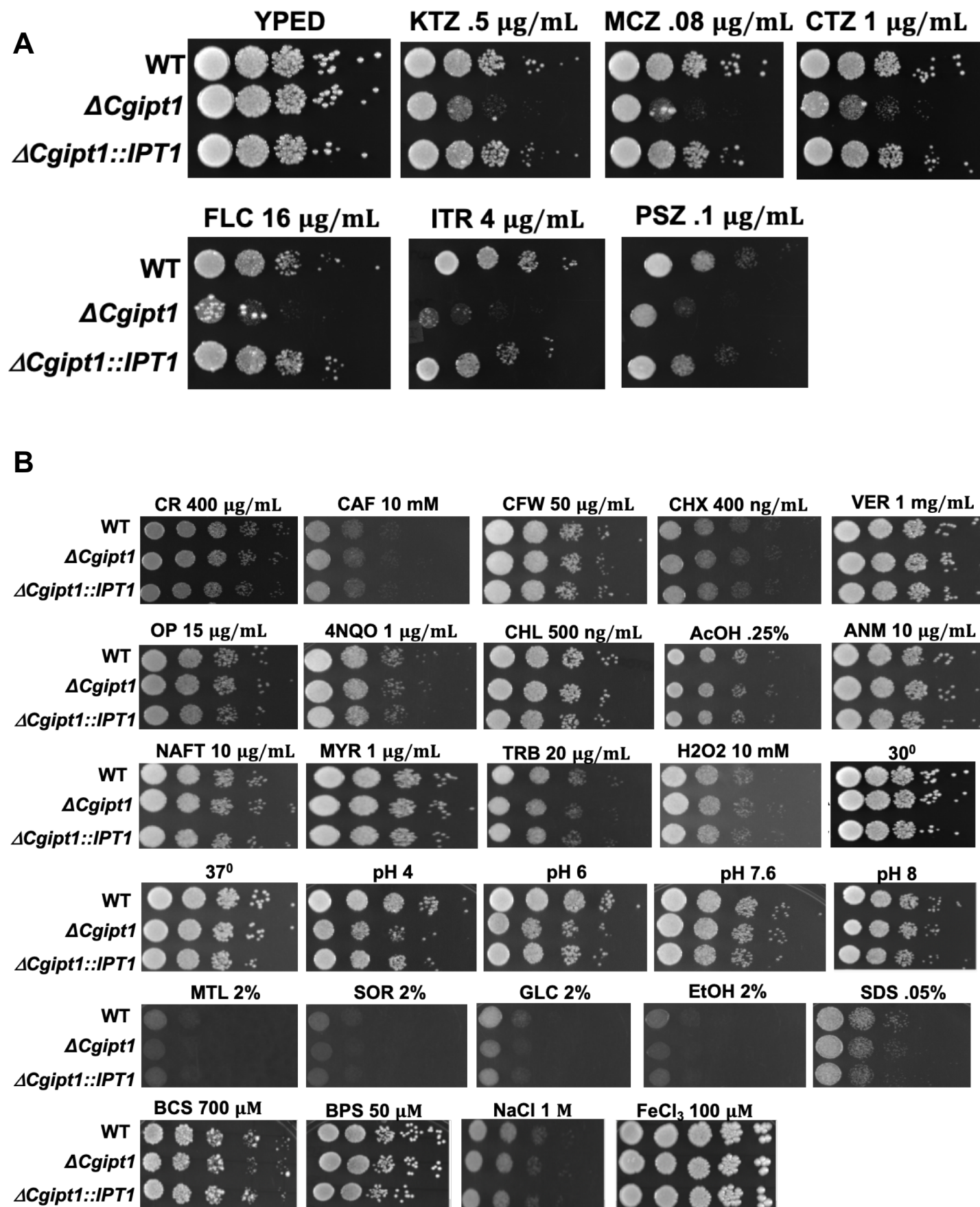

Supplement: Supplementary file 1 [file jof-08-00651-s001.zip › jof-1775877-supplementary/Figure S1.pdf]

**Figure S2**

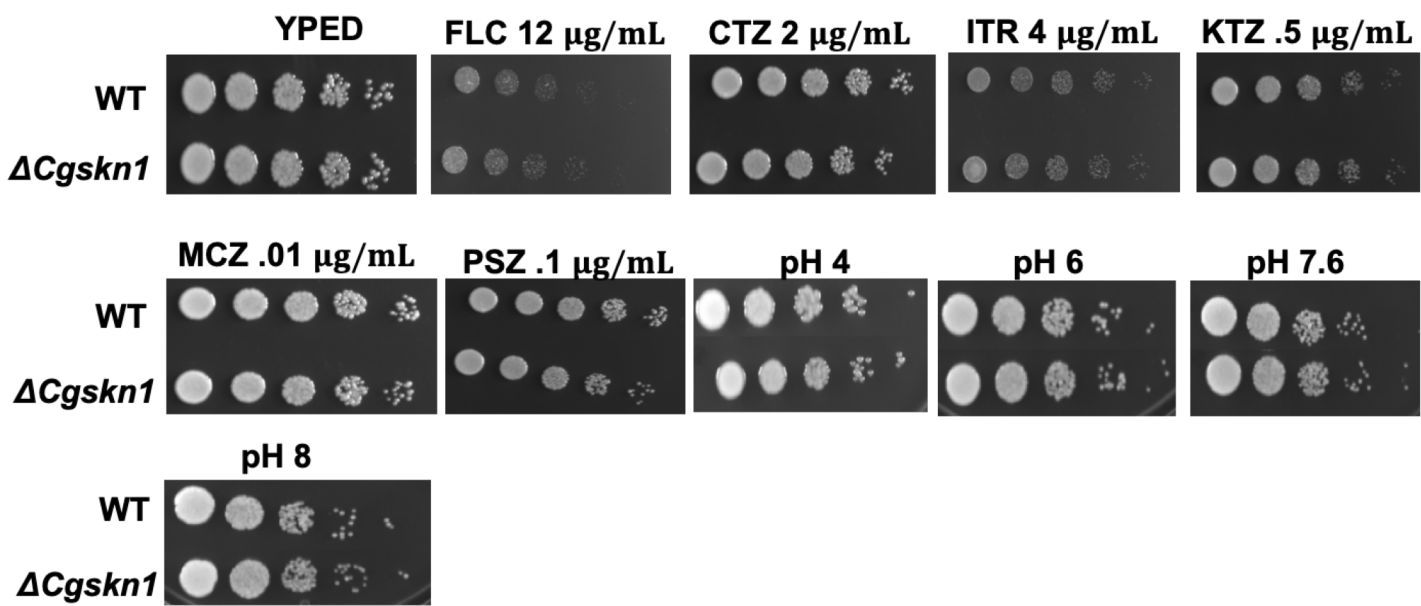

Supplement: Supplementary file 1 [file jof-08-00651-s001.zip › jof-1775877-supplementary/Figure S2.pdf]

**Figure S3**

**A**

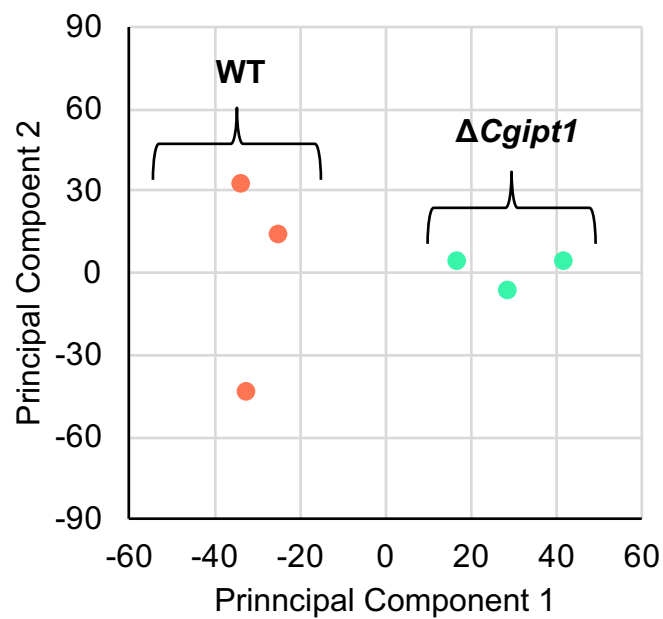

**B**

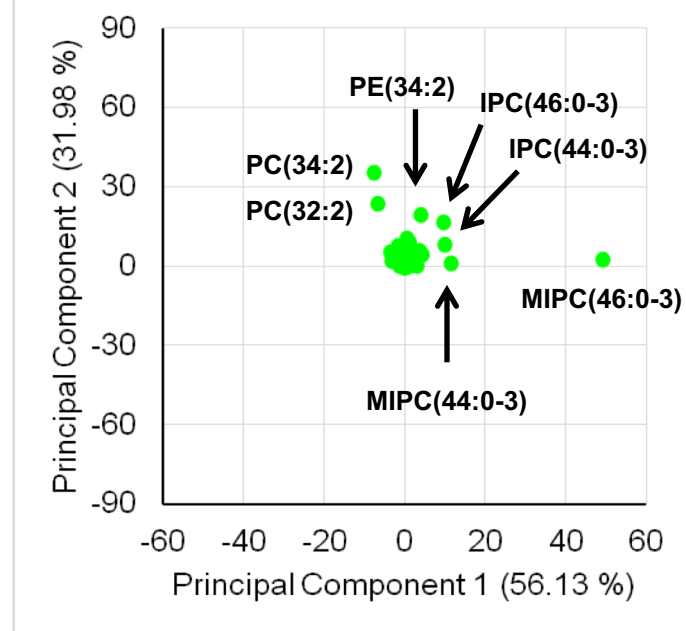

Supplement: Supplementary file 1 [file jof-08-00651-s001.zip › jof-1775877-supplementary/Figure S3.pdf]
